# Improved Pharmaceutical Formulæ

**Published:** 1872-12

**Authors:** 


### IMPROVED PHARMACEUTICAL FORMULÆ.

The following improved formulæ are given by Mr. J. Donde, in the American Journal of Pharmacy, February, 1872:

### SYRUP OF RHATANY.

| | |
|---|---|
| Dry Extract of Rhatany, - - - | 3 ounces. |
| Rain Water, - - - - - - | 5 ounces. |
| Simple Syrup, - - - - - | 15 fluid ounces |

Pulverize the extract, mix it with the water and 5 ounces of the syrup in a capsule, which place in a steam-bath, occasionally stirring with a glass rod until the extract is dissolved; add the rest of the syrup, and evaporate to 18 fluid ounces. Each fluid drachm contains 10 grains of extract.

When sulphuric lemonade is prescribed with extract of rhatany it is necessary to add a little gum syrup to prevent the precipitation of the extract.

### SYRUP OF VALERIAN TO REPLACE INFUSIONS.

| | |
|---|---|
| Hydro-alcoholic Ext. of Valerian Root, - | 2 ounces, |
| Rain Water, - - - - - - | 2 ounces, |
| Simple Syrup, - - - - - | 4 fl'd ounces. |

Concentrate the syrup to 32 deg. Bme.; when cooled to 40 or 50 deg. C., add the extract dissolved in the water. When cold it has 36 deg. Bme. Each fluid ounce contains 18 grains of extract, equivalent to 1 drachm of the root. One fluid ounce of this syrup with 8 of water may advantageously replace the infusion.

Would it not be convenient to employ syrups for preparing drinks which are now made by infusion or decoction? There would result the advantage of having the medicine whenever needed, and always with the same proportion of medicinal principles.

The fluid extracts would be very useful in the preparation of these syrups.

### CHALK MIXTURE.

Mr. Geo. W. Kennedy, in the same journal, recommends the following changes in this preparation. He says he finds the ordinary mixture to become sour and mouldy in warm weather.

By way of experiment in order to obviate this great inconvenience I tried the substitution of glycerin for sugar, and so far, up to the present time, I have found it to work well after the following formula:

R.—Cretæ Præpt., - - - -
Glycerinæ (Bower's), - - aa oz. ss.
Pulv. Acaciæ, - - - - dr. ij.
Olei Cinnamoni, - - - - gtt. viij.
Aqæ Destill., - - - - oz. viij.

Mix thoroughly.

The above mixture I have kept a whole summer and up to the present time; I made it about ten months ago, and upon opening it I found it in perfect condition, not even the slightest acidification having taken place.

The above process is not used for dispensing chalk mixture in my shop, but was only tried by way of experiment, to see if it would keep during the hot summer months.

The following formula has been used by me for some time back:

R —Cretæ Præpt., - - - - oz. ss.
Sacchari Albi., - - - - -
Pulv. Acaciæ, - - - - aa dr. ij.
Olei Cinnamoni, - - - gtt. viij.

Mix intimately.

For every fluid ounce of chalk mixture I take one drachm of the mixed powders, and rub them well up with an ounce of distilled water, and of course the mixture is free from acidity. In cases of diarrhœa in children, which generally is the result of fermentation, the glycerin formula seems to be preferable to the one containing sugar, the former mixture being and remaining bland, nutritious and with soothing effect on the bowels; to a certain extent it arrests fermentation, and the glycerin fully protects the gum from decomposition.

### SOAP LINIMENT.

Mr. J. A. Graefle (same journal), recommends the following formula:

R.—Take of dry white Castile soap (finely grated), oz. iv.
Camphor, - - - - - - oz. ij.
Oil of Rosemary, - - - - fl. oz. ss.
Water, - - - - - - fl. oz. vj.
Alcohol, - - - - - - fl. oz. xxx.

Put the soap in a half-gallon bottle, pour on a pint of alcohol, shake well, add the water and shake again till the soap is dissolved.

Dissolve the camphor and oil in the remaining alcohol, mix the two solutions and filter.

From five to ten minutes is all that is necessary for the solution of the soap, and the resulting liniment, when finished, is beautifully clear.

Mr. R. Rother writes to the Chicago Pharmacist, December, 1871:

| | |
|---|---|
| R.—Take of soap (genuine Castile, mottled or white) dry and in No. 12 powder, . . | 24 troy ounces |
| Camphor, . . . . . . . . . . . . . | 12 troy ounces |
| Oil of Rosemary, . . . . . . . . | 3 fluid ounces |
| Water, . . . . . . . | 3 pints. |
| Strong Alcohol, . . . . . | 10½ pints. |

Mix the water with half a pint of the alcohol in a capacious vessel; add the soap and apply heat until the solution has occurred; to this add four pints of alcohol. In the remaining six pints of alcohol dissolve the camphor and oil; to this add the solution of soap; mix. Let the impurities (coloring matter of the soap), subside and filter.

